# Medical Society of the State of California

**Published:** 1856-12

**Authors:** 


### *Medical Society of the State of California.*

We have been favored by a friend with the proceedings of the Convention, and of the Medical Society of the State of California, held in Sacramento, March, 1856. An invitation was addressed to the members of the profession throughout the State, which resulted in the assembling of about seventy-five physicians, representing sixteen counties, for the purpose of forming a State Society. The convention elected B. F. Keene, M. D., of El Dorado, President.

The principle objects accomplished by this meeting were, *first*, the formation of a State Society, with a Constitution and By-Laws. *Second*, The adoption of the Code of Ethics of the American Medical Association.

A good primary medical education attested by a diploma from some known medical school of good repute ; and a good professional and moral standing in the community, are conditions for admission into the State Society. A committee was appointed to prepare a bill and urge its passage, aiming to give legal recognition to the medical profession of the State, and as far as possible, to protect the people from empiricism.

Provision was made for the establishment of a Medical Journal, to be edited by Dr. J. F. Morse, of Sacramento.

A resolution was adopted commending a proper devotion of medical men to the interests of the profession, and expressing a "sovereign contempt for that species of professional mountebankery that seeks to secure public favor and pecuniary advantage, by foisting upon public attention, through newspapers and otherwise, the peculiar qualifications of their author to treat particular diseases, either in the department of medicine or surgery."

Standing Committees were appointed as follows:

On *Practical Medicine*, *Medical Literature and Hygiene.*

On *Surgery.*

On *Obstetrics.*

On *Medical Topography*, *Meteorology*, *Endemics and Epidemics.*

On *Indigenous Botany*, *or Domestic Adulteration of Drugs.*

On *Medical Education.*

On *Publication.*

A *Committee of Arrangements.*

A resolution was passed inviting members to propose subjects of scientific interest for discussion by the Society.

Also, one recommending the profession in the different counties of the State to organize County Societies, for the purpose of co-operating with the State Society in promoting the interests of the profession.

A Committee was appointed on Prize Essays. Measures were adopted to collect Zoological, Botanical and Mineralogical specimens.

We have thus given an imperfect outline of the doings of our brethren in California. We can not too heartily commend their zeal and interest in relation to professional matters. The tone and general bearing of the proceedings would do honor to much older communities.

It will be seen that the excellent Code of Ethics of the American Medical Association has been adopted.

We predict for the Society great usefulness, and that it will be an honor to the State, and that the profession will soon be enriched with valuable contributions from that far off region.—*Cincinnati Observer.*

